# Developing a Process for the Analysis of User Journeys and the Prediction of Dropout in Digital Health Interventions: Machine Learning Approach

**DOI:** 10.2196/17738

**Published:** 2020-10-28

**Authors:** Vincent Bremer, Philip I Chow, Burkhardt Funk, Frances P Thorndike, Lee M Ritterband

**Affiliations:** 1 Institute of Information Systems Leuphana University Lüneburg Lüneburg Germany; 2 Center for Behavioral Health & Technology University of Virginia School of Medicine Charlottesville, VA United States

**Keywords:** dropout, digital health, machine learning

## Abstract

**Background:**

User dropout is a widespread concern in the delivery and evaluation of digital (ie, web and mobile apps) health interventions. Researchers have yet to fully realize the potential of the large amount of data generated by these technology-based programs. Of particular interest is the ability to predict who will drop out of an intervention. This may be possible through the analysis of user journey data—self-reported as well as system-generated data—produced by the path (or journey) an individual takes to navigate through a digital health intervention.

**Objective:**

The purpose of this study is to provide a step-by-step process for the analysis of user journey data and eventually to predict dropout in the context of digital health interventions. The process is applied to data from an internet-based intervention for insomnia as a way to illustrate its use. The completion of the program is contingent upon completing 7 sequential cores, which include an initial tutorial core. Dropout is defined as not completing the seventh core.

**Methods:**

Steps of user journey analysis, including data transformation, feature engineering, and statistical model analysis and evaluation, are presented. Dropouts were predicted based on data from 151 participants from a fully automated web-based program (Sleep Healthy Using the Internet) that delivers cognitive behavioral therapy for insomnia. Logistic regression with L1 and L2 regularization, support vector machines, and boosted decision trees were used and evaluated based on their predictive performance. Relevant features from the data are reported that predict user dropout.

**Results:**

Accuracy of predicting dropout (area under the curve [AUC] values) varied depending on the program core and the machine learning technique. After model evaluation, boosted decision trees achieved AUC values ranging between 0.6 and 0.9. Additional handcrafted features, including time to complete certain steps of the intervention, time to get out of bed, and days since the last interaction with the system, contributed to the prediction performance.

**Conclusions:**

The results support the feasibility and potential of analyzing user journey data to predict dropout. Theory-driven handcrafted features increased the prediction performance. The ability to predict dropout at an individual level could be used to enhance decision making for researchers and clinicians as well as inform dynamic intervention regimens.

## Introduction

The efficacy of digital (ie, internet, web, and mobile) behavioral interventions to improve a range of health-related outcomes has been well documented [1-3]. However, adherence to these interventions is a significant issue [4]. Intervention dropout, defined as a participant prematurely discontinuing a program, from internet-based treatments for psychological disorders typically varies between 30% and 50% [4-6]. However, the reason for such high dropout rates is still unclear [5], whereas longer treatment duration and user engagement appear to be associated with improved treatment outcomes and greater effectiveness of the digital intervention [7-10]. Furthermore, in a research setting, high dropout rates and, consequently, low exposure to digital content might affect the reported effects of a digital intervention and the validity of the results [11,12]. Although researchers have highlighted the need for a science of user attrition [13], there have been few advances in predicting dropout through advanced quantitative approaches in eHealth interventions [14]. In particular, previous work has identified hypothetical factors influencing attrition in eHealth programs, such as ease of leaving the intervention, unrealistic expectations on behalf of users, usability and interface issues, and amount of workload required to benefit from an intervention [13]. Such factors are likely to impact how a user ultimately engages with a program and could provide indicators for predictive factors but do little to advance predictive modeling of dropout when not applied in data-driven studies. Research suggests that an increased completion of modules in digital therapeutics increases treatment outcomes [15]. Identifying those patients that are likely to drop out of treatment and addressing the related issues can, thus, improve treatment outcomes and can be the basis of the development of micro interventions that target these high-risk participants to reengage them to complete the program [16]. Thus, predicting dropout on a participant level supports the decision making of experts in the target field and consequently leads to more personalized treatment strategies. In addition, inferential results can increase insight into the causes of attrition by revealing data-driven indicators. Participant-specific factors can help to identify individuals who benefit more from digital therapies compared with individuals for whom face-to-face treatment might be a better approach. To evaluate the possibility of predicting dropout in digital interventions and to shed light on some indicators of dropout, the aim of this study is to propose a process for user journey analysis to predict dropout from a digital intervention.

A wealth of data can be collected through the use of digital interventions. They often feature content that is administered over time as users complete tasks or components of the intervention, typically over several weeks or months [17-20]. Digital interventions also track and log different types of user interactions (eg, frequency of log-ins). These data provide a nuanced understanding of the usage behavior of participants over the course of an intervention [21]. Combined with self-reported data, passively collected user data could be captured and used to provide deeper insight into how likely users are to drop out of an intervention on an individual level and lead to increased prediction performance.

A user journey is a sequence of interactions as an individual uses a digital intervention (ie, the path an individual takes to navigate through a program). Although user journeys are well known and established in the field of web-based marketing, to the best of our knowledge, its direct application to digital health interventions has not yet been examined. Web-based marketers leverage user journeys to collect information about an individual’s behavior [22], often referred to as clickstream data analysis [23,24]. This increases the understanding of users’ behavior by recognizing patterns in their sequence of actions. Thus, user journey analysis can reveal insight into an individual’s behavior by enabling an analysis of data (eg, Ecological Momentary Assessment [EMA] or log data) that is not frequently used in the eHealth sphere [25].

There are several possible reasons why analysis of user journeys has not achieved prominence in digital health interventions. One obstacle lies in the analysis of large amounts of raw data. Analysis of user journeys often requires transformation of raw data, feature engineering, and the application of machine learning techniques, which can be a burdensome process [26] and is not a typical skill set of eHealth behavior researchers. Although user journeys have been used to predict different psychological factors such as mood, stress levels, or treatment outcomes and costs [25,27-31], to our knowledge, no work has provided steps to be taken to analyze raw user journey data and, at the same time, predict user dropout from a digital health intervention.

The overarching goal of this study is to establish and provide a step-by-step process that describes how to leverage user journeys to predict various behaviors (eg, dropout). This process involves several steps, including creating the basic data structure for handling user journeys, creating features that can add additional information to the existing raw data, and ultimately providing a framework for the statistical analysis. A technical implementation (R package) [32,33] of this process is provided for the research community. To demonstrate the application and potential utility of this process, we use it to predict user dropout in a randomized controlled trial of a fully automated cognitive behavior therapy intervention for insomnia (Sleep Healthy Using the Internet [SHUTi]) [34].

## Methods

### User Journey Process

**Figure 1:**
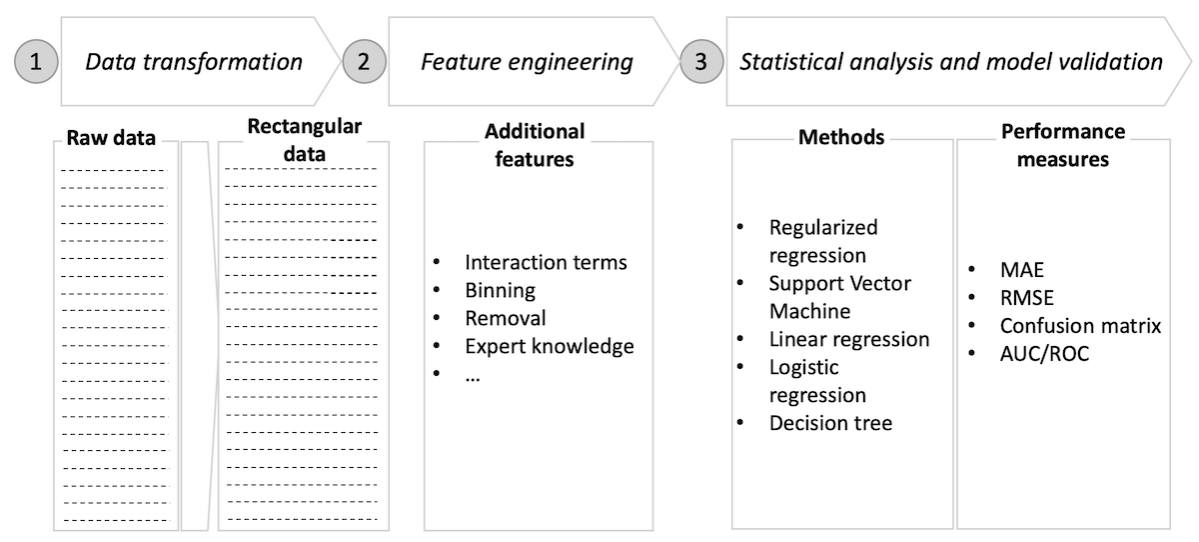
Process of analysis. AUC: area under the curve; MAE mean absolute error; ROC: receiver operating characteristics; RMSE: root mean square error.

The overarching steps of the user journey process are outlined in Figure 1. This process applies machine learning algorithms, specifically supervised learning, which is used when both input (eg, log-ins and mood symptoms) and output data (eg, dropout status) exist in the data set [35].

It is important for researchers to clearly define the outcome variable of interest. As dependent variables can take on different measurement scales (eg, discrete or continuous), defining the target variable has consequences for the choice of statistical models. When predicting discrete outcomes (ie, consisting of at least two discrete categories or labels), classification is often the appropriate approach. However, when predicting continuous outcome variables, the learning task is regression.

#### Step One: Data Transformation

**Figure 2:**
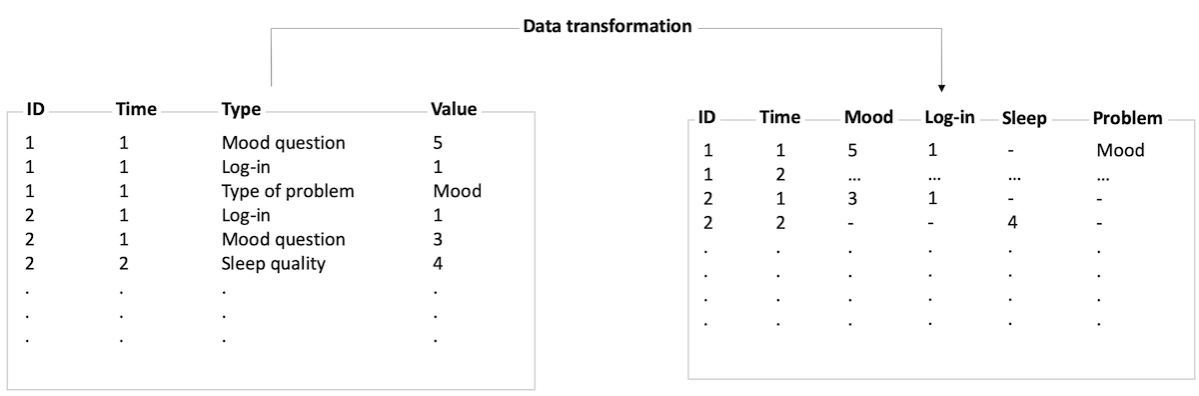
Example of data transformation in the context of digital health interventions.

The first step in analyzing user journey data is to transform the raw data into a wide format, as can be seen in Figure 2. Thus, the transformed data are structured such that each row corresponds to a unique observation in *Time* for a particular user (*ID*).

When transforming the raw data, it is important to specify the time window defining the time interval for which individual touch points are aggregated. The choice of the time window depends on the density of the observations in the raw data. For example, if a raw data set is composed of a few touch points over the course of a day, choosing a time window on a scale of days avoids sparseness of the transformed data matrix. In contrast, when predicting purchases in web-based marketing, for example, a large number of observations exist for each user on short timescales. Here, choosing a small window (eg, an hour) could be beneficial, as the resulting matrix will not be sparse and information loss is minimal. In an internet-based intervention, however, it is not unusual for self-reported data to be collected as little as once a day, with a user logging into the system only a few times a day. In this case, it would not make sense to choose an hour-long window because the resulting matrix would be very sparse. Thus, choosing a time window on a scale of days would be a better choice.

If multiple observations of the same type occur within a time window, one must decide how to aggregate these values. For some variables, such as diary entries, taking an average may be desirable; for other variables, such as log-ins, the sum is a more appropriate aggregation. The provided technical framework supports the data transformation procedure. In addition, missing values often exist in the data. There are various procedures that can handle missing values. One might remove all rows that include missing values; however, this can lead to a reduction in observations. Other possibilities include imputation procedures such as using aggregated values of these features or developing statistical models that predict the missing values based on other features. For more information on missing values, we refer to the study by Batista and Monard [36].

#### Step Two: Feature Engineering

Feature engineering can be described as the process of including additional variables into the data with the intention of achieving increased predictive performance. As statistical learning relies heavily on the input data, this step is important for improving the accuracy of prediction [37]. There are 2 approaches to feature engineering: handcrafted or automated. Handcrafted feature engineering is a challenging task and requires human effort and domain knowledge. Therefore, it is appropriate for researchers with expertise in the domain that is represented by the data (eg, sleep) to be highly involved in the process [38-40]. A clear understanding of the problem to be solved is necessary to derive meaningful features [40]. Handcrafted feature engineering often involves a trial and error phase to experiment with different features [37]. Automated feature engineering involves the generation of candidate features that are evaluated based on their predictive performance. Tools exist for the application of automated feature engineering in different domains, such as natural language processing or machine vision [38,41,42].

Interaction terms, that is, the product of 2 original features, can lead to additional knowledge about their relationships and increased predictive accuracy. The provided technical framework supports generating them. In case of a large number of original features, however, including interaction terms results in many additional features.

**Figure 3:**
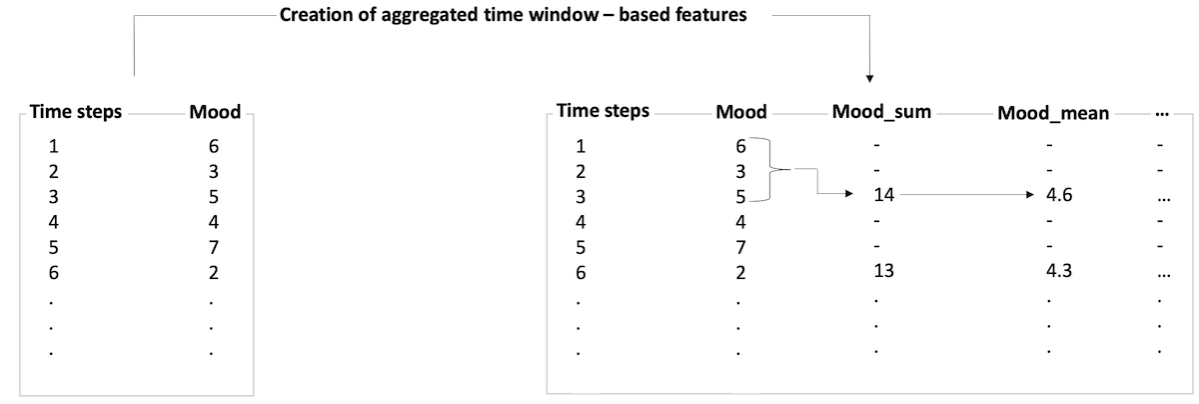
Example of creating aggregated time window–based features for w=3.

In addition, time window–based aggregation methods can be beneficial in terms of predictive performance in the context of digital health interventions [31]. Here, based on a user-specified time window *w*, various types of aggregations are performed on the original features. Figure 3 represents the process of this task through the exemplification of self-reported EMA data. The *Mood* level is reported by an individual at different points in time (*Time steps*). For the creation of the aggregated features, a time window of *w*=3 is specified in this example. Various statistical measures, such as the sum (*Mood_sum*), mean (*Mood_mean*), minimum, maximum, and SD (not shown in figure), are calculated for 3 consecutive measurements of the mood level (*w*=3) and included as additional features in the data set. It should be noted that the creation of features can limit one’s ability to reproduce study results if the feature engineering process is not well documented or if the data set changes over time. For the case study in this paper, we created various theory-driven features based on expert knowledge, which will be introduced in *Feature Engineering*.

#### Step Three: Statistical Analysis and Model Validation

**Figure 4:**
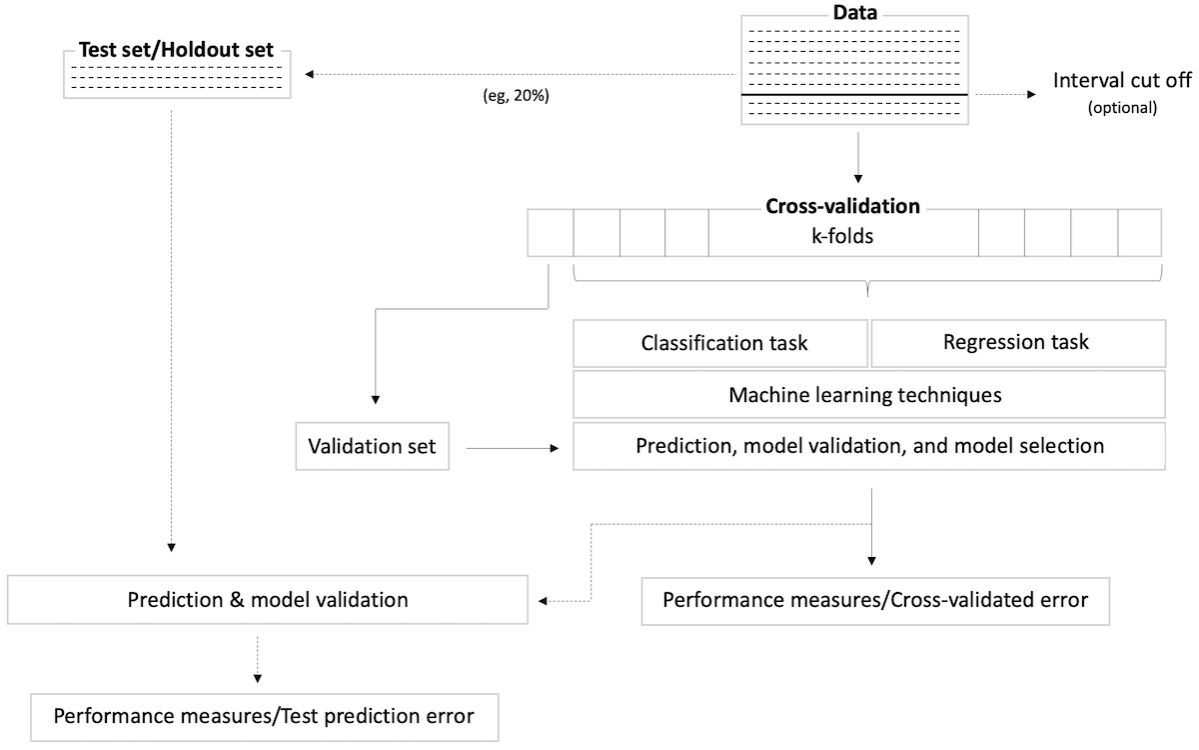
Procedure of statistical analysis.

The next step in analyzing user journey data is the application of machine learning techniques to predict the outcome variable. Figure 4 depicts this procedure. First, the data set can be split into a training set for fitting the data and learning patterns and a test (or holdout) set. This test set is usually created if sufficient data are available. It is subsequently used to test the final model performance of the selected algorithm. It is difficult, however, to quantify *sufficient data* as it depends strongly on the field of research, applied models, and structure of the data.

Depending on the task to be analyzed, the data can be further split based on particular points in time. If the aim of the analysis, for example, is the prediction of the outcome of an intervention, it might be useful to evaluate at what point in time the predictive accuracy is at its peak. The longer the time window, the higher the predictive accuracy can be assumed because more data are available. Thus, using time windows and basing the amount of usable data on these windows (*interval cut off*) can be useful in evaluating the feasibility of prediction.

There are a large number of machine learning techniques that can be applied to user journey data; some models can be applied to both learning tasks (classification or regression), such as support vector machines or decision trees, whereas others fit better for a specific task (ie, logistic regression for classification). Researchers may wish to compare their predictive performance to justify the model selection. Cross-validation is often applied to gauge the predictive performance of a specified model. Here, the data are divided into k chunks, where k-1 chunks are used for training the machine learning techniques and the remaining data chunk is used for predicting the target variable. This procedure is repeated k times until each chunk has been used as a validation set. Ultimately, the model with the best performance is selected for the specified learning task. If a holdout set is maintained, the specified model is then trained based on all data. The target variable in the holdout set is then predicted and evaluated, which leads to the test prediction error.

Model validation checks the ability of a particular model to either fit the data or predict the outcome variable [43]. Eventually, the one with the best performance is selected. Nonvalidation can lead to inaccurate predictions and, thus, overconfidence in the developed model [44]. Model validation should generally be executed on the validation set for each iteration of the cross-validation procedure (cross-validated prediction error) to select the best model and, subsequently, on an independent test set that was set aside earlier (test prediction error). In some cases, especially when sufficient data are not available, no independent test set is put aside and only the cross-validated error is reported, which can lead to an optimistic estimation of the error [44].

Deciding on the method of model validation also depends on the learning task. For regression, criteria such as the root mean square error or mean absolute error are often appropriate. For the classification task, confusion matrices and receiver operating characteristic (ROC) graphs are often used as performance indicators. More information about these validation procedures and their application can be found elsewhere [45].

In the provided technical framework, logistic regression, linear regression, support vector machines, boosted decision trees, and regularization techniques are implemented. As overfitting can occur when utilizing a large number of features [37] and some types of statistical procedures (eg, linear regression) cannot be applied when the number of features is greater than the number of observations, alternative techniques such as regularization and feature selection may need to be used [46]. A thorough review of these techniques is outside the scope of this paper, and readers are strongly encouraged to learn more about each of these techniques and how they pertain to their data and aims.

### Case Study

To illustrate the user journey analysis process, data were extracted from a trial of a web-based program (SHUTi) [47]. SHUTi is a fully automated web-delivered program that is tailored to individual users [47] and informed by the model for internet interventions [17]. SHUTi is based on the primary principles of face-to-face cognitive behavioral therapy for insomnia (CBT-I), including sleep restriction, stimulus control, cognitive restructuring, sleep hygiene, and relapse prevention. SHUTi contains 7 *cores* that are dispensed over time, the first core being a tutorial on how to use the program, with new cores becoming available 7 days after completion of a previous core. This format was meant to mirror traditional CBT-I delivery procedures using a weekly session format. SHUTi has been found to be more efficacious than web-based patient education in changing primary sleep outcomes (insomnia severity, sleep onset latency [SOL], and wake after sleep onset [WASO]), with the majority of SHUTi users achieving insomnia remission status 1 year later [48]. A mobile app version of SHUTi, Somryst, with equivalent content and mechanisms of action was recently cleared by Food and Drug Administration as the first prescription digital therapeutic for treating patients with chronic insomnia. Thus, the efficacy of SHUTi is well established. However, similar to other digital interventions, predicting user dropout is an important yet unaddressed issue. Thus, the primary aim of this case study is to demonstrate the feasibility of predicting user dropout from data generated by a digital health intervention.

The sample for this study was drawn from a trial consisting of 303 participants (218/303, 71.9% female) aged between 21 and 65 years (mean 43.3 years, SD 11.6). They were 83.8% (254/303) White, 6.9% (21/303) Black, 4.0% (12/303) Asian, and 5.3% (16/303) *other*. Participants were randomly assigned (using a random number generator) to receive SHUTi or web-based patient education (control condition). The study was approved by the local university’s institutional review board, and the project was registered on clinicaltrials.gov (NCT01438697). Inclusionary and exclusionary criteria as well as outcomes are reported in detail elsewhere [48].

Data from 151 participants who were assigned to SHUTi were used in this study. Both self-reported and system-generated types of data are available. Participants completed a battery of self-report measures at baseline and post intervention. A list and detailed description of the measures have been published previously [48]. Sleep diaries were also collected throughout the intervention period, along with information about bedtime, length of sleep onset, number and duration of awakenings, perceived sleep quality, and rising time. Data were collected prospectively for 10 days (during a 2-week period) at each of the 4 assessment periods (pre- and postintervention and 6- and 12-month follow-ups). Sleep diary questions mirrored those from the consensus sleep diary [49]. Values for SOL and WASO were averaged across the 10 days of diary collection at each assessment period. The system-generated data included individual log-ins and automated emails sent by the system as well as trigger events logged in the system. All data were used to predict user dropout, defined as not completing all 7 SHUTi cores (core 0 through core 6). Thus, users were classified as having dropped out or not. As noted elsewhere [48], 60.3% (91/151) participants completed all 7 cores in the SHUTi program.

## Results

**Figure 5:**
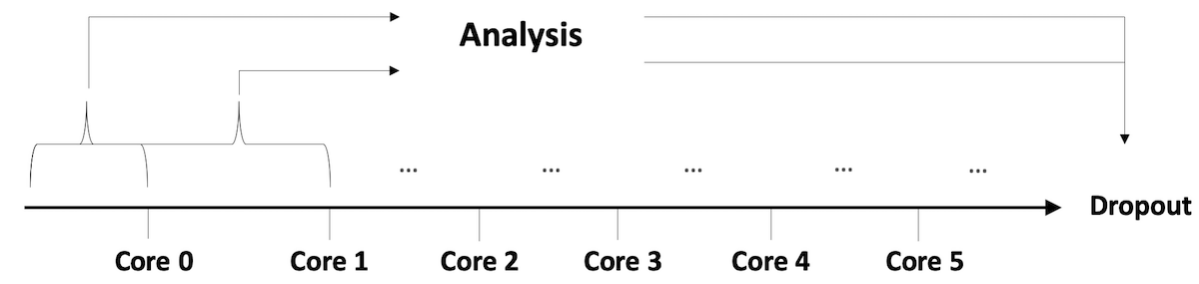
Setup of analysis for dropout prediction.

The primary aim was to predict whether users prematurely dropped out of SHUTi (dropped out by core 6/completed core 6). Therefore, the learning problem is a binary classification (drop out/did not drop out). To verify the point at which the machine learning techniques were capable of predicting dropout, separate analyses were executed after the completion of each core (Figure 5) and only included data up to the core in question. The number of participants included in each analysis was 146, 141, 133, 116, 102, and 101 for cores 0 to 5, respectively.

### Data Transformation

As a first step, the raw data were transformed into a rectangular data matrix (wide format), which led to 981 basic features. Basic features are those features that were already included in the raw data. As an example, see column *Type* in Figure 2. In addition, 25 handcrafted and theory-driven features that were derived from the raw data were implemented. These features are introduced in the next section *Feature Engineering*. In total, 1006 features were used for the analyses. Whenever the same question (ie, in the case of diary data) was administered multiple times a day, the mean of the reported values was chosen for numeric data and the mode for categorical data. To reduce the sparseness of the resulting data matrix, reported values for questionnaires such as the Insomnia Severity Index were repeated for each participant until the next occurrence of the questionnaire (this questionnaire was administered before each core). To address the issue of missing data, features were deleted based on the quantity of missing data. To evaluate how the deletion affects the predictive performance of the models, features were deleted that contained more than 5%, 10%, 15%, and 20% of missing values. This procedure reduced the number of features tremendously. In addition, categorical variables that had only one level or category were removed. Less data are available for the analysis at time point core 0 compared with time point core 5. Thus, the number of features for each level of missing data was 83, 263, 299, and 401 features.

As the aim of this study was to predict dropout at core 6, each participant only had exactly one outcome value—they could either complete core 6 or not. Users that dropped out between cores 1 to 5 would be classified as having dropped out at core 6. Therefore, the user journey data must be aggregated for each user. For most of the variables, the mean and mode were used as the aggregation method. However, for some variables, such as log-in information or number of days since the last contact, the sum is more appropriate. Table 1 illustrates the different aggregation procedures and the corresponding features. Features that are not listed were aggregated by mean and mode. The rest of the missing data were imputed using the median for numeric variables and mode for categorical features. In addition, an imputation based on the k-nearest neighbor (KNN) algorithm was applied (k=5). Both approaches were used to reveal which of them led to a better prediction performance.

**Table 1:** Aggregation of theory-determined features.

Feature aggregation method	Handcrafted features	Existing clinically important features
Sum: The sum of all observations of a specific feature for an individual	Days since the last contact (any interaction) If sleeping duration is decreasing from core to core If sleep window duration is 5 or 8 hours	If the participant had an alcoholic drink that day If the participant took a nap If the system recorded a triggered event that day If the participant logged in that day If the system sent an email that day
Last: The last observation of a specific feature for an individual	Difference between preferred arising time in core 2 and core 3 If preferred arising time is greater than 8 AM in core 2 Average time in days to complete a core among all cores that have been available Time needed in days to complete a core in days (6 features for core 0-5)	If the participant finished homework in core 2 Number of days where no diaries have been completed in the period of analysis Precipitating factor includes *major life event* or *health/psychological*
Mean: Mean of the observations of a specific feature for an individual	Difference between awake and arise time Difference between preferred arise time and actual arise time (AM/PM) Difference between preferred arise time and actual arise time (minutes) Difference between preferred bedtime and actual bedtime	Naptime in minutes

### Feature Engineering

A total of 25 theory-driven features were implemented for this case study. Some of these features, shown in Table 1, were handcrafted and some were already existing in the data set. Specifically, the handcrafted features were computed from the raw data and were deemed useful for model prediction. Few of these features are study-specific (eg, *if the participant finished homework in core 2*), whereas others could be used in any type of digital intervention (eg, *if the participant logged in*). As the number of features generated from the study data was already large, none of the generic feature generation methods were used. These 25 features were not deleted based on the missing value ratio (mentioned above) because there was a clinical or theory-driven rationale that they would influence prediction performance.

### Statistical Analysis and Model Validation

**Figure 6:**
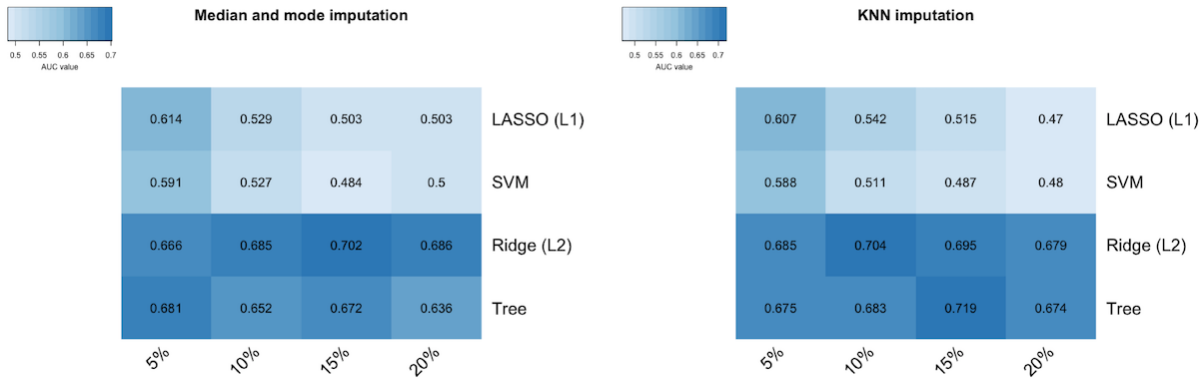
Heat map of average area under the curve values across core analyses for each model, imputation procedure, and threshold for percentage of missing values. AUC: area under the curve; KNN: k-nearest neighbor; LASSO: least absolute shrinkage and selection operator; SVM: support vector machine.

For the learning task, a set of machine learning techniques was used to select the model with the best prediction performance. Specifically, support vector machines, boosted decision trees, and logistic regression with L1 and L2 regularization were applied. The optimal parameters were determined using a grid-based search and cross-validation. In addition, stratified 10-fold cross-validation was used for each analysis. To choose an appropriate statistical model, a heat map was created to illustrate the average area under the curve (AUC) across all core analyses for each model, imputation procedure, and threshold for percentage of missing values (Figure 6). As can be seen, the method of imputing the missing values did not have a strong influence on the performance of the applied statistical model. Increasing the percentage threshold negatively influenced L1 regularization and the support vector machine, whereas L2 regularization and boosted decision trees seemed not to be influenced tremendously. The best average AUC value (0.719) was achieved by applying boosted decision trees, deleting each feature that contained more than 15% of missing values, and imputing the rest of the missing values by KNN.

**Figure 7:**
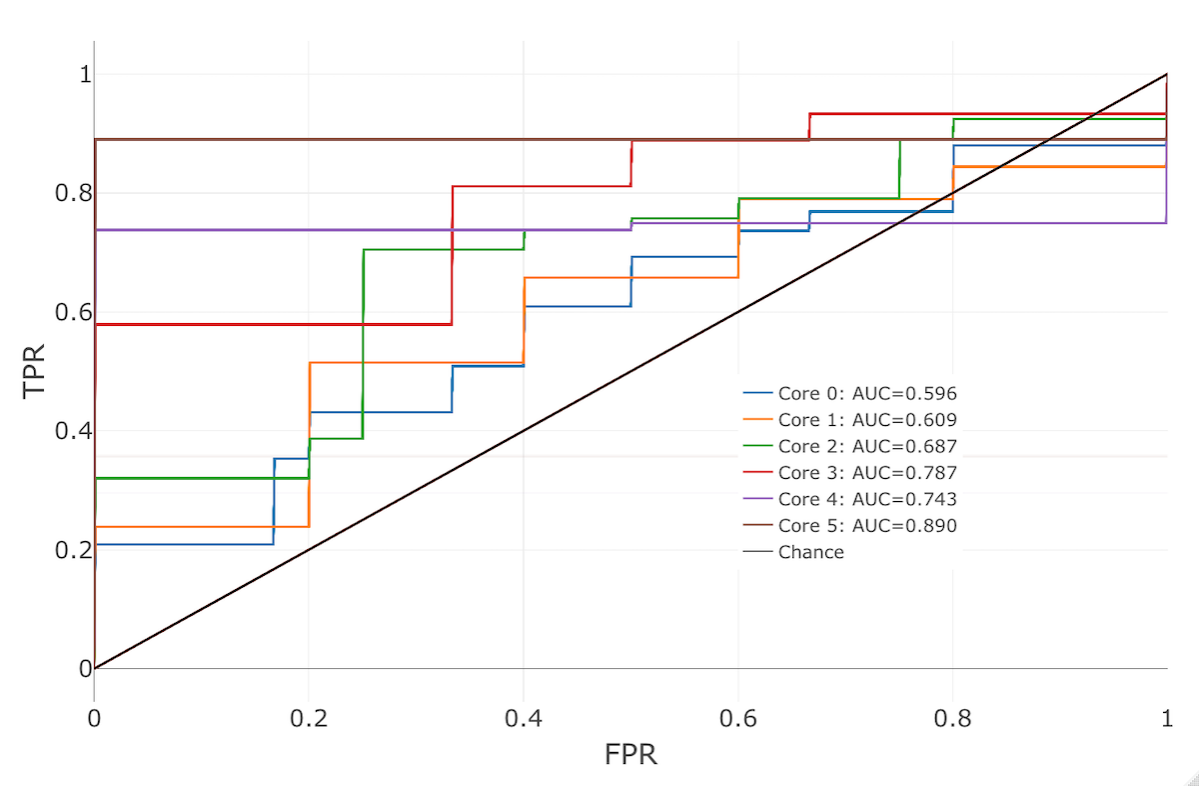
Receiver operating characteristic for each core analysis based on boosted decision trees (15% missing value deletion, k-nearest neighbor imputation). AUC: area under the curve; FPR: false-positive rate; TPR: true-positive rate.

Figure 7 illustrates the ROC curves for each core analysis using the specified parameters. With the exception of core 4, the AUC values increased with each analysis. For each core, the predictions were better than random, indicated by AUC values above 0.5. Generally, the AUC values ranged between 0.6 and 0.9. Importantly, the prediction of dropout appears feasible early in the intervention period (ie, core 1 and core 2). In addition, the area under the precision-recall curve (PRAUC) was computed. Across all core analyses, a PRAUC of 0.48 was observed, whereas chance had an average of 0.24. Thus, the model performs better than chance.

Boosted decision trees were used to identify important features. Here, SHapley Additive exPlanation (SHAP) values were used [50]. SHAP values are a relatively new concept in the field of machine learning and essentially represent the importance of each feature and their contribution to the prediction by comparing the prediction of the model with and without a specified feature value depending on the order of their introduction to the model. In addition to the importance of each feature, SHAP values quantify how features contribute to the prediction of the model.

**Figure 8:**
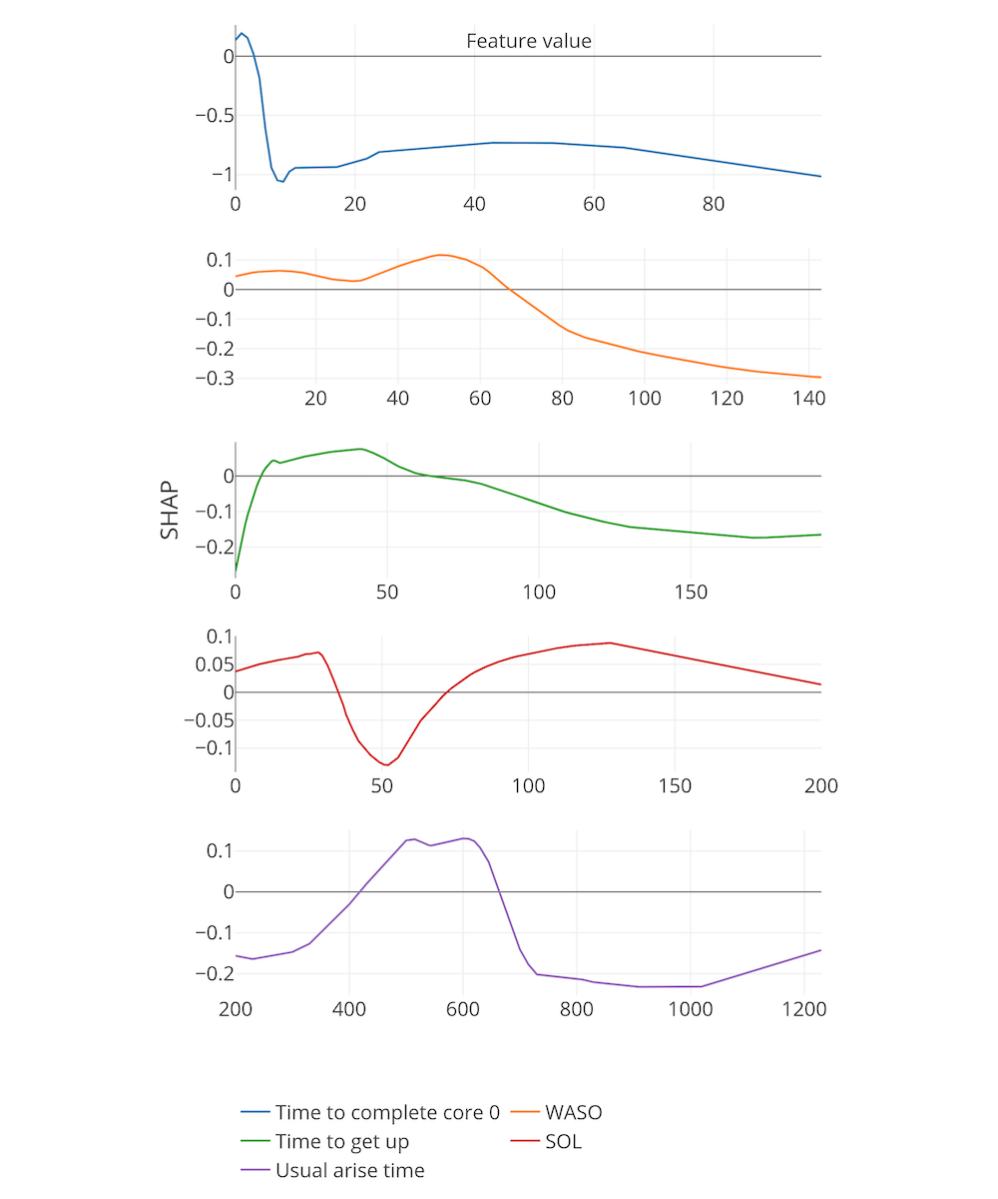
Five most important features for each core analysis according to boosted decision trees (15% deletion of missing values, and k-nearest neighbor imputation). The x-axis represents the values for each feature, and the y-axis represents the SHAP values. SHAP: SHapley Additive exPlanation; SOL: sleep onset latency; WASO: wake after sleep onset.

**Figure 9:**
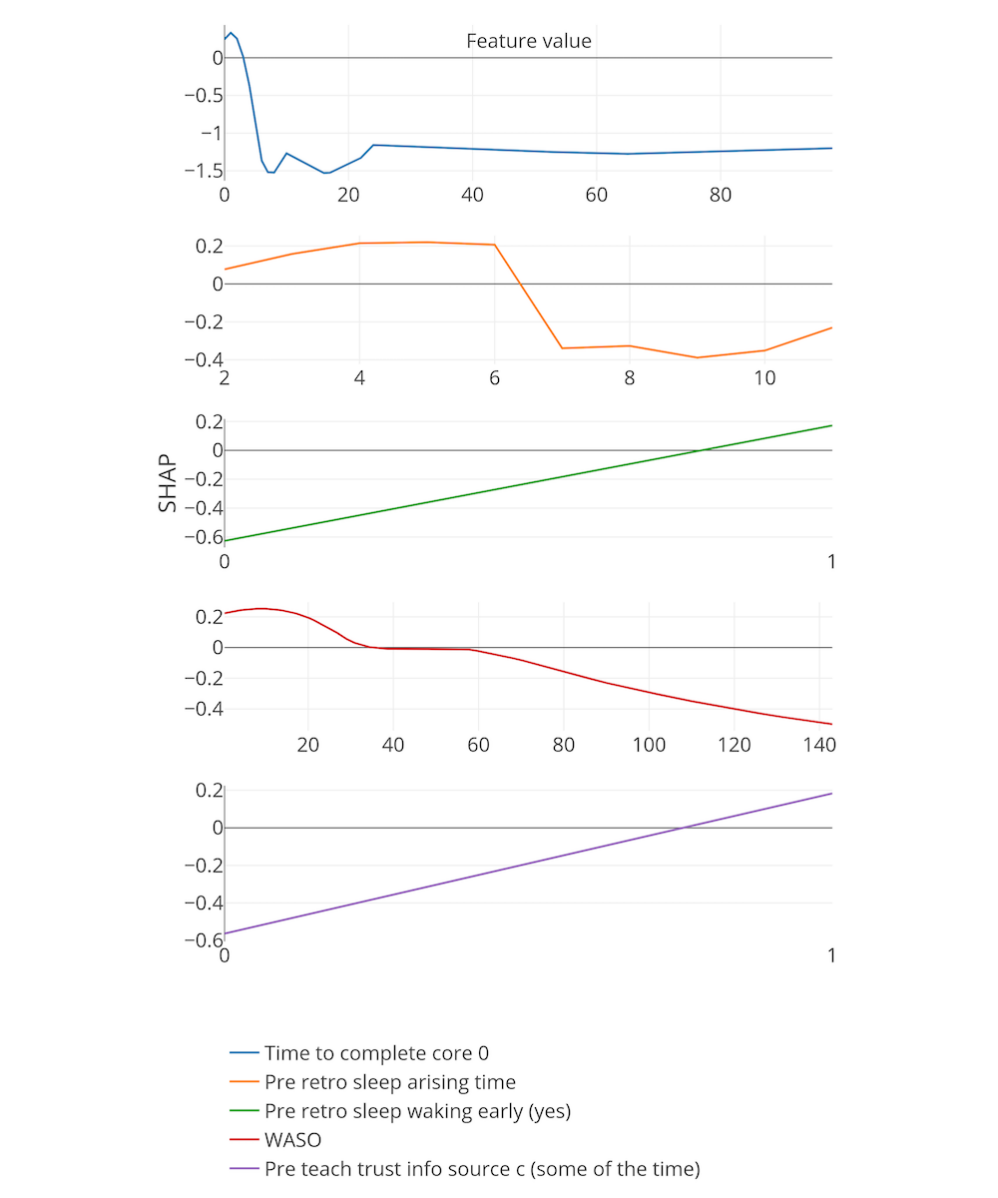
Five most important features for each core analysis according to boosted decision trees (15% deletion of missing values, KNN imputation, and Core 1 analysis). SHAP: SHapley Additive exPlanation; WASO: wake after sleep onset.

**Figure 10:**
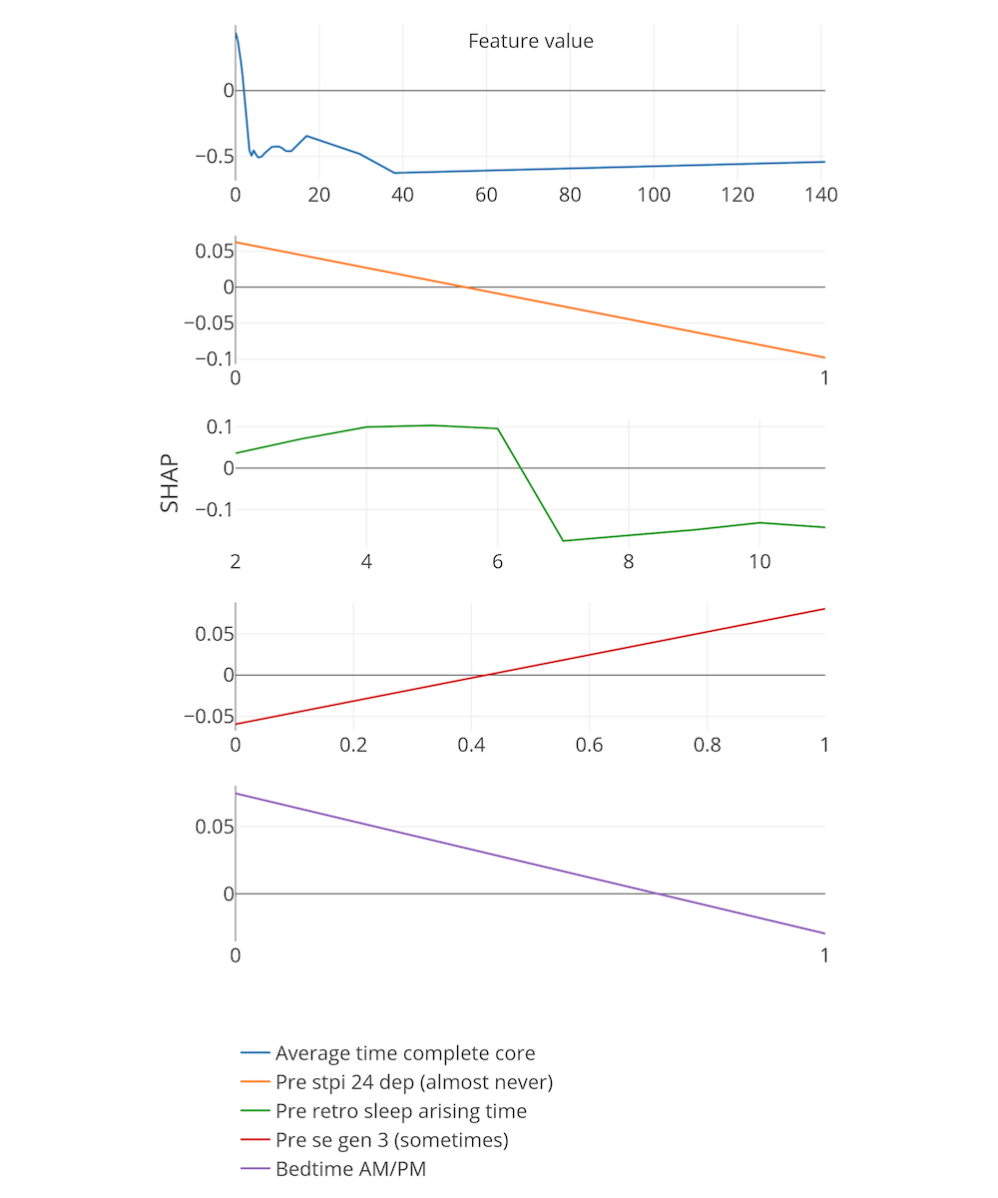
Five most important features for each core analysis according to boosted decision trees (15% deletion of missing values, KNN imputation, and Core 2 analysis). SHAP: SHapley Additive exPlanation.

**Figure 11:**
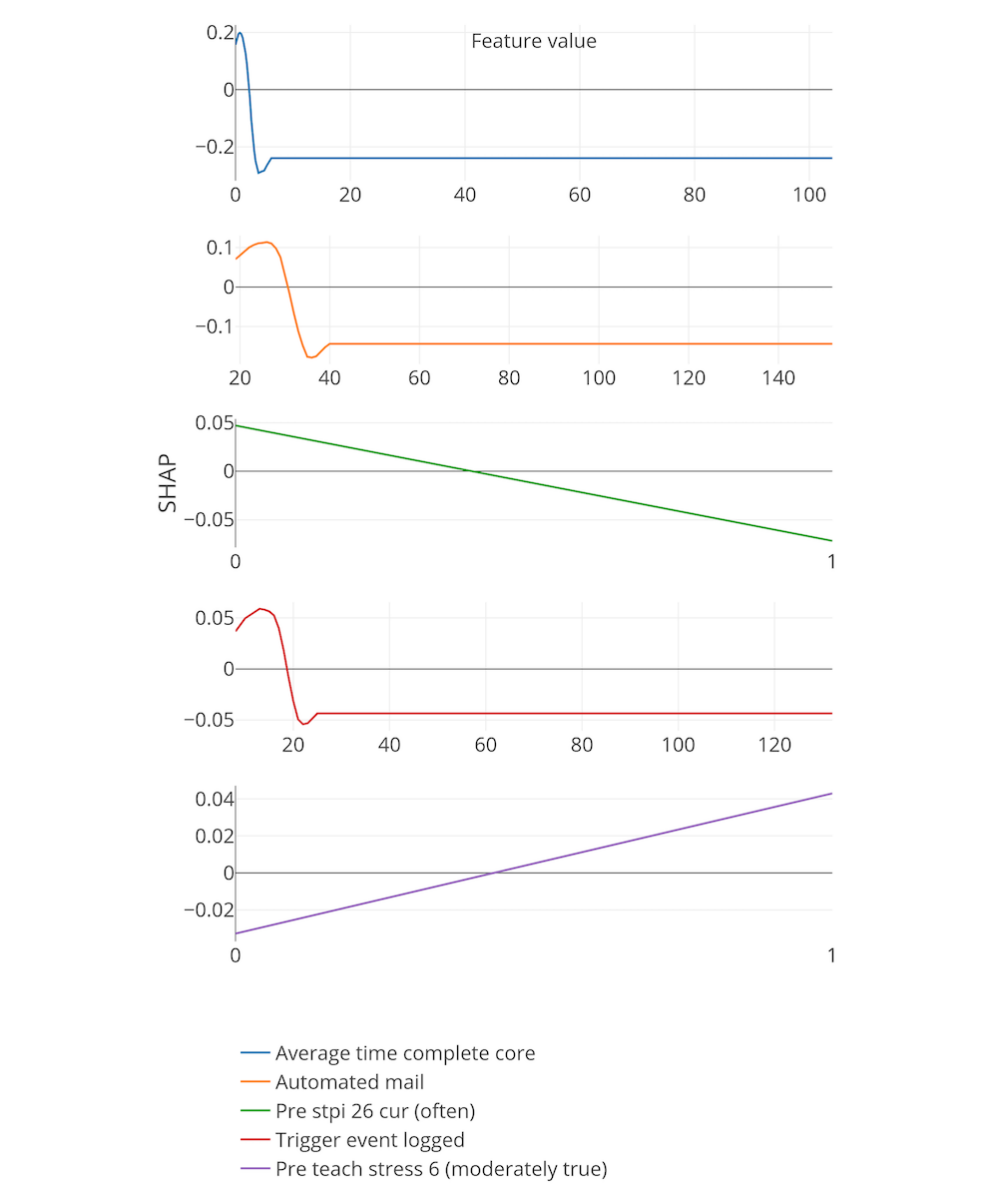
Five most important features for each core analysis according to boosted decision trees (15% deletion of missing values, KNN imputation, and Core 3 analysis). SHAP: SHapley Additive exPlanation.

**Figure 12:**
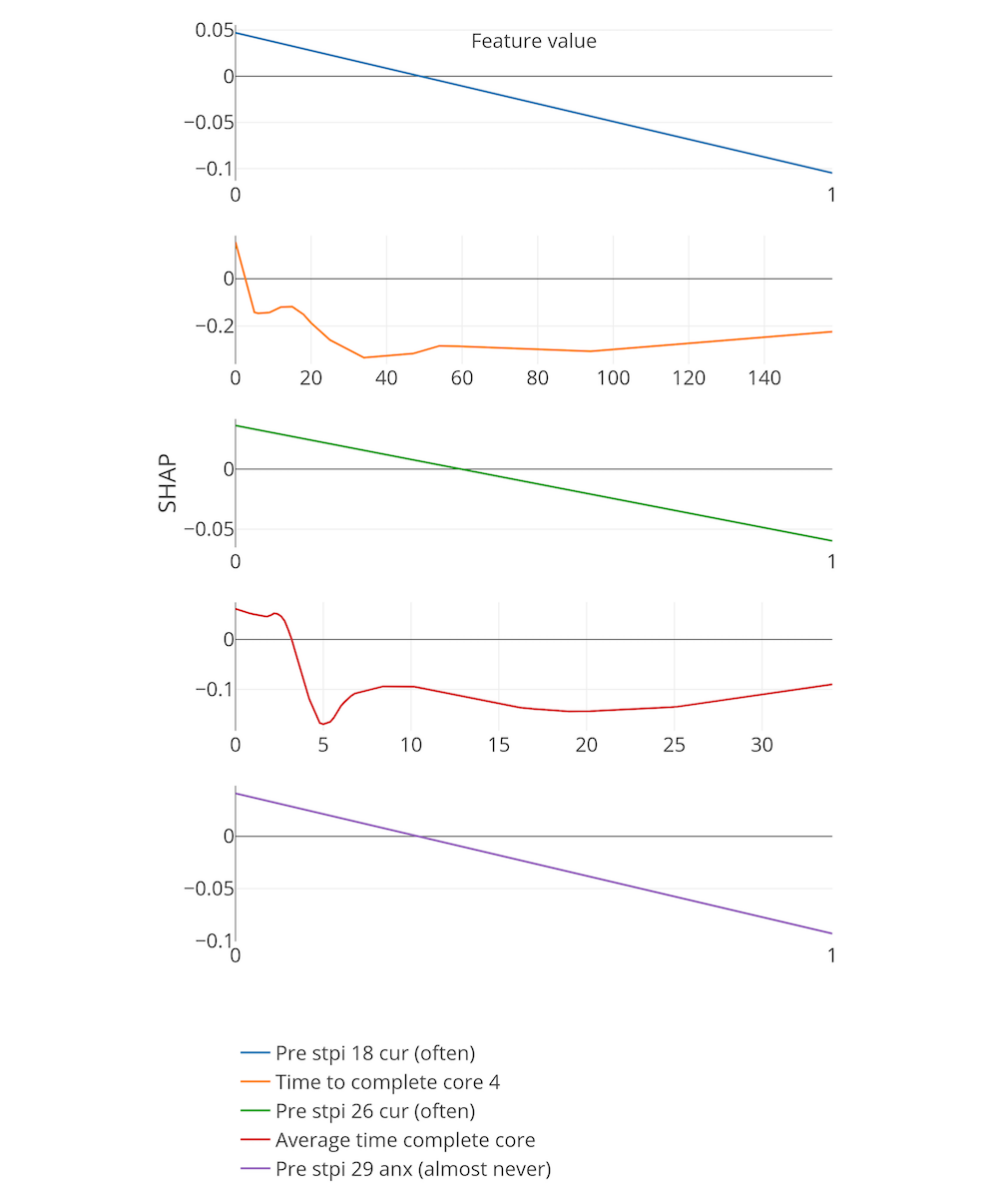
Five most important features for each core analysis according to boosted decision trees (15% deletion of missing values, KNN imputation, and Core 4 analysis). SHAP: SHapley Additive exPlanation.

**Figure 13:**
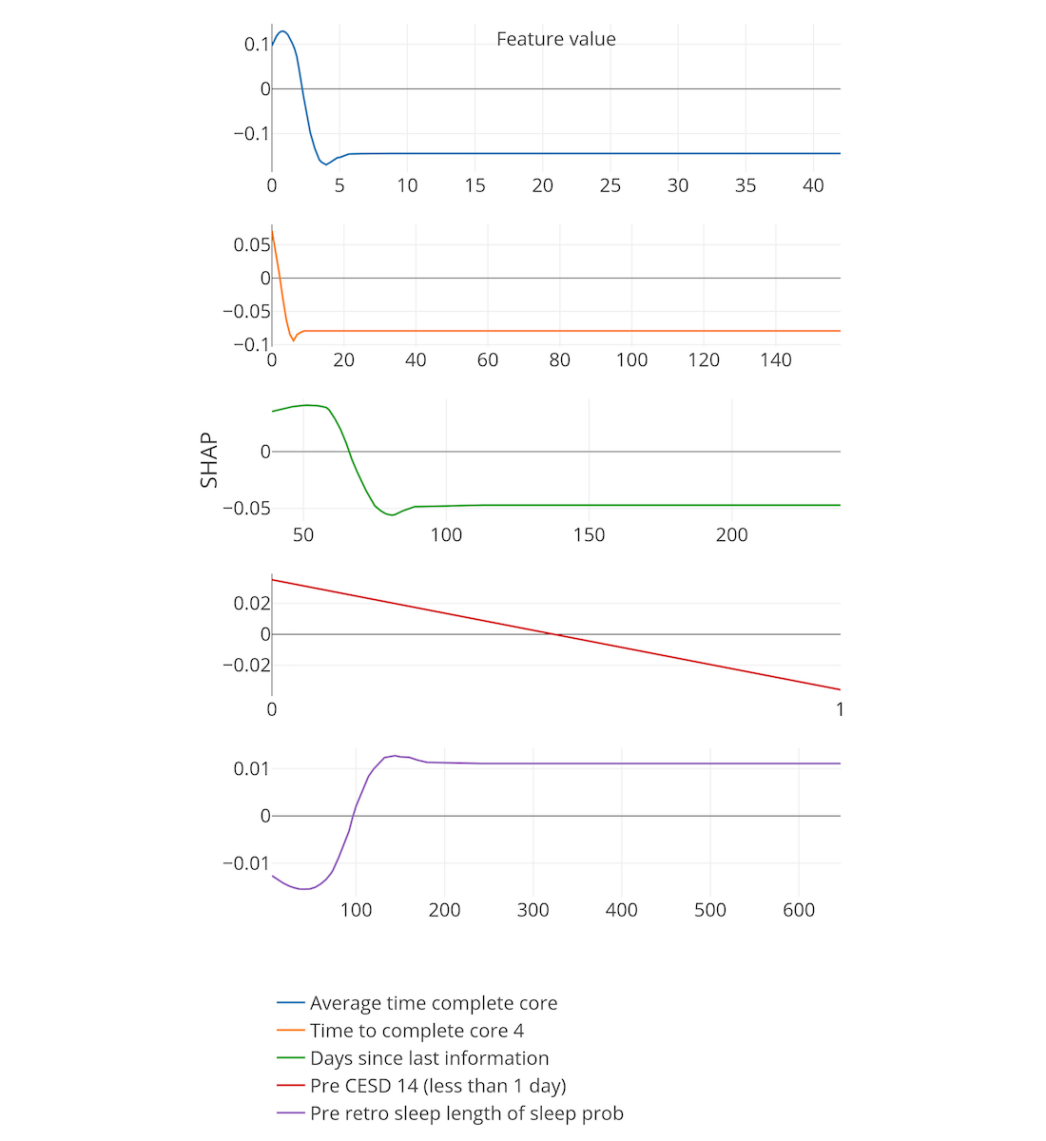
Five most important features for each core analysis according to boosted decision trees (15% deletion of missing values, KNN imputation, and Core 5 analysis). SHAP: SHapley Additive exPlanation.

Figures 8-13 include the 5 most important features according to the boosted decision trees for each core analysis. In each graph, the x-axis represents the values for each feature and the y-axis represents the SHAP values (ie, the effect each feature has on predicting the completion of core 6 of the intervention). In the core 0 analysis, for example, finishing core 0 within 3 days (x-axis) has a positive influence on dropout, as can be seen on the y-axis above zero. However, taking more time to complete core 0 (where x-axis is greater than 3) influences dropout prediction negatively as the graph approaches values under zero.

In general, 7 out of the strongest 22 features were handcrafted and theory driven. Table 2 summarizes all the features. Taking more time to complete the cores appeared to influence dropout. The time to complete core 0 predicted whether a participant eventually dropped out (core 0 and core 1 analysis). In addition, usual arise time and the time needed to get out of bed (from awake to arise) affected the prediction of dropout early on in the intervention. Participants who got up earlier than 4:30 AM and later than 6:45 AM, and participants who needed less than 9 min or more than 66 min to get up, negatively influenced the prediction of completing core 6 of the intervention (x-axis of the feature usual arise time and time to get up for core 0). Furthermore, a greater WASO also appeared to influence the prediction of dropout status. These variables could, therefore, be an early indicator of dropout in this particular intervention.

In addition, if triggers were logged on for more than 18 days or participants received emails for more than 30 days, dropping out was more likely (core 3 analysis). Furthermore, if there was no interaction between the system and the participants for more than 67 days, the individuals were more likely to drop out.

**Table 2:** Summary of the unique top 5 most important features across analyses.

Predictors		Analysis at each point in time					
Feature	Description	Core 0	Core 1	Core 2	Core 3	Core 4	Core 5
Core 0 completion date—intervention start date^a^	Time to complete core 0 in days	+^b^	+	N/A^c^	N/A	N/A	N/A
Arise time—awake time^a^	Difference between time of awakening and getting out of bed in minutes (time to get up)	+	N/A	N/A	N/A	N/A	N/A
Usual arise time	Retrospective report specified from baseline data	+	N/A	N/A	N/A	N/A	N/A
Wake after sleep onset	Minutes awake in the middle of the night from sleep diaries	+	+	N/A	N/A	N/A	N/A
Sleep onset latency	Minutes to fall asleep from sleep diaries	+	N/A	N/A	N/A	N/A	N/A
Baseline arise time (pre retro sleep arising time)	Time the user specified that they got out of bed from baseline data	N/A	+	+	N/A	N/A	N/A
Pre retro sleep waking early	User indicates having problems waking up too early in the morning	N/A	+	N/A	N/A	N/A	N/A
Pre teach trust info source c	How much the user trusts health information	N/A	+	N/A	N/A	N/A	N/A
Average time to complete core^a^	Average time to complete a core among all cores that have been available up to the point of the analysis	N/A	N/A	+	+	+	+
Pre stpi 24 dep^d,e^	How low the user feels at baseline	N/A	N/A	+	N/A	N/A	N/A
Pre se gen 3^f^	How well the user feels things have been going	N/A	N/A	+	N/A	N/A	N/A
Bedtime	If a participant went to bed in the AM or PM (before or after 12 AM)	N/A	N/A	+	N/A	N/A	N/A
Email sent^a^	If the system sent an email that day	N/A	N/A	N/A	+	N/A	N/A
Pre stpi 26 cur^g^	How stimulated the user feels at baseline	N/A	N/A	N/A	+	+	N/A
Trigger event logged^a^	If the system logged a trigger event that day	N/A	N/A	N/A	+	N/A	N/A
Pre teach stress 6	User feels he or she can solve most problems if necessary effort is put in	N/A	N/A	N/A	+	N/A	N/A
Pre stpi 18 cur^h^	How eager the user feels at baseline	N/A	N/A	N/A	N/A	+	N/A
Core 4 completion date—core 4 start date^a^	Time to complete core 4 in days	N/A	N/A	N/A	N/A	+	+
Pre stpi 29 anx^i^	How much self-confidence the user feels at baseline	N/A	N/A	N/A	N/A	+	N/A
Days since the last information^a^	Days since the last contact (any interaction)	N/A	N/A	N/A	N/A	N/A	+
Pre CESD^j^ 14^k^	How lonely the user feels at baseline	N/A	N/A	N/A	N/A	N/A	+
Pre retro sleep length of sleep prob	Number of months the user reports having had sleep difficulties at baseline.	N/A	N/A	N/A	N/A	N/A	+

^a^Handcrafted/theory-driven features.

^b^+ indicates appearance of feature in corresponding core analysis.

^c^N/A: not applicable.

^d^STPI: state-trait personality inventory.

^e^Pre stpi 24 dep: baseline STPI measure item #24 depression subscale.

^f^Pre se gen 3: baseline Perceived Stress Scale item #5.

^g^Pre stpi 26 cur: baseline STPI measure item #26 curiosity subscale.

^h^Pre stpi 18 cur: baseline STPI measure item #18 curiosity subscale.

^i^Pre stpi 29 anx: baseline STPI measure item #29 anxiety subscale.

^j^Center for Epidemiologic Studies Depression Scale.

^k^Pre CESD 14: baseline CESD measure item #14.

## Discussion

### Principal Findings

Considering the increasing use of digital health interventions and the tremendous amount of data gathered in such interventions, a variety of methods can be used for the analysis of various data types and structures. In this study, a process for the analysis of user journey data in this context was proposed, and a step-by-step guide and technical framework for the analysis as an R package was provided. Challenges of data analysis based on user journeys, such as data transformation, feature engineering, and statistical model application and evaluation, were discussed. The analysis of user journeys can be a powerful tool for the prediction of various factors on an individual participant level. Here, it has been applied to real-world data to predict dropout from an internet-based intervention.

The application of the proposed process and evaluation of statistical models indicated the feasibility of dropout prediction by using this process. AUC values ranged between 0.6 and 0.9 for the selected machine learning algorithm (boosted decision trees). Most importantly, it was shown that the prediction of user dropout was possible early in the intervention, which could be helpful to clinicians and policy makers as treatment decisions are made and adjusted. In addition, this study indicated the importance of expert knowledge and subsequent implementation of handcrafted features. Not all existing statistical models necessarily require handcrafted features because automated feature engineering can already provide crucial insight; however, handcrafted features can increase prediction performance and lead to increased interpretability. In this study, handcrafted features appeared to be among the most important features according to the boosted decision trees, perhaps given the more nuanced understanding necessary for treating insomnia. It is important to keep in mind, though, that the analysis presented here was meant as a demonstration of the power of this approach. A much larger data set is needed to draw more firm and generalizable conclusions.

With this caveat, a number of interesting results emerged related to features and impact on dropout prediction. For example, as participants took longer to complete earlier steps of the intervention, they were less likely to complete the final step of the intervention. Thus, a discussion about how users can be motivated to complete early steps in the intervention may be very beneficial. In addition, the findings suggest that the time participants get out of bed in the morning and how much time they actually needed to get up might be an important factor for completing the sleep intervention. Participants who get out of bed between 4:30 AM and 6:45 AM and do not need more than 66 min to get out of bed were more likely to complete the final step of the intervention. In addition, trigger events might only have a positive effect in the short term, as the appearance of triggers more often than 18 days appeared to increase the likelihood of dropping out. However, it could be possible that this finding only accounts for participants who would not have completed the final step of the intervention. Assuming this, these participants were, therefore, not influenced by trigger events. It is also important to emphasize that these results are based on a bottom-up, data-driven learning approach. Therefore, it is up to researchers to interpret the results and cross-validate them in other samples. Predictions in this context based on user journey data and the resulting knowledge about factors that influence these predictions, especially on an individual level, could lead to the implementation of strategies that seek to improve the utilization and efficacy of digital health interventions.

### Limitations

There are a number of limitations of this study that should be considered when interpreting the results. One limitation is the relatively limited number of participants included in the analysis and the large feature space. The predictive performance of the applied models is satisfactory, especially early on in the intervention. The process and models described in this study are technically feasible, although the reliability of the ensuing results may be impacted by limitations to sample size. Owing to the limited number of participants, the results of this study should be replicated in a larger sample. Furthermore, the amount of missing values impacts the analyses and can lead to bias. Obtaining more complete data can further increase the interpretability and predictive accuracy of the models. In addition to time window–based features and time-dependent variables, the demonstrated steps and this study in general do not include time-dependent feature engineering, such as the relation between features and observations across time. Researchers should examine the data set they are planning to analyze to determine whether time-dynamic features could be used in their projects. Another limitation is the fact that the data are heterogeneous at an individual participant level; thus, the application of models that consider heterogeneous parameters might provide deeper and more individualized information about the participants. However, considering the number of participants in the data, heterogeneous models have not yet been investigated. The results are, nevertheless, promising and can lead to increased knowledge about users and how dropout from digital health interventions is affected by various factors. Studies using larger data sets are necessary to improve model performance and confirm findings.

### Conclusions

This study proposes a step-by-step process for the analysis of user journey data in the context of digital health interventions and provides a technical framework. Furthermore, the proposed framework was applied to data from an internet-based intervention for insomnia to predict dropout of participants. These participants needed to complete 7 cores to finish the program. Importantly, our process was able to predict user dropout at each core better than chance. The predictive performance also varied by core; although the AUC was approximately 0.6 for cores 0 and 1, it was noticeably higher for the latter cores. This indicates that the user journey process can be used to predict dropout early in the intervention and prediction accuracy increases over the course of the intervention. This may allow researchers to preemptively address dropout before it occurs by providing support to users that may be struggling to engage. Among the machine learning techniques we evaluated, boosted decision trees provided the greatest accuracy while deleting features that contained more than 15% missing values. In addition, a varying set of features was revealed that contributed to the prediction performance of dropout in this context. Replicating the results of this study in a larger sample is needed to further validate the process outlined in this paper. Researchers may also wish to develop methods that predict the likelihood of user dropout over the duration of an intervention, which could enable researchers to devote resources to those at the highest risk of dropping out.

## Abbreviations

AUC: area under the curve
CBT-I: cognitive behavioral therapy for insomnia
EMA: Ecological Momentary Assessment
KNN: k-nearest neighbor
PRAUC: area under the precision-recall curve
ROC: receiver operating characteristic
SHAP: SHapley Additive exPlanation
SHUTi: Sleep Healthy Using the Internet
SOL: sleep onset latency
WASO: wake after sleep onset
